# Etoricoxib Induced Toxic Epidermal Necrolysis in a Case of Systemic Lupus Erythematosus: A Case Report

**DOI:** 10.31729/jnma.7665

**Published:** 2022-09-30

**Authors:** Asim Pandey, Samriddhi Parajuli, Alok Dhungel, Rahul Devkota, Angel Dangol

**Affiliations:** 1 Kathmandu Medical College and Teaching Hospital, Sinamangal, Kathmandu, Nepal; 2 Department of Internal Medicine, Kathmandu Medical College and Teaching Hospital, Sinamangal, Kathmandu, Nepal

**Keywords:** *case reports*, *etoricoxib*, *systemic lupus erythematosus*, *toxic epidermal necrolysis*

## Abstract

Toxic epidermal necrolysis is a potentially life-threatening dermatological condition whose pathogenesis and exact treatment are not yet known. Drugs like anticonvulsants, allopurinol and nonsteroidal anti-inflammatory drugs like etoricoxib, a selective cyclo-oxygenase-2 inhibitor prescribed for pain management are associated with a high risk of toxic epidermal necrolysis. It is also associated with immunodeficiency and dysregulated immune reactions like systemic lupus erythematosus, an autoimmune disease in which organs and cells undergo damage initially mediated by tissue binding auto-antibodies and immune complexes. Here, a 34 year old lady was presented in emergency with multiple maculopapular rashes over the neck and trunk region after treatment with etoricoxib for osteoarthritis of the left foot.

## INTRODUCTION

Toxic epidermal necrolysis (TEN) is an unpredictable, life-threatening cutaneous drug reaction accounting for 77-95% of cases.^1,2^ Patient presents with acute onset painful skin lesions, fever, sore throat and conjunctivitis, and epidermal detachment of >30% body surface area (BSA). A study done in china showed that the incidence of TEN was 0.4 to 1.2 per million but Asian patients were at two-fold risk.^3^ This is a case of 34 years old female who presented with a maculopapular rash over the neck and trunk region after treatment with etoricoxib for osteoarthritis of the left foot.

## CASE REPORT

A 34 years old female presented to the emergency department with multiple itchy maculopapular rashes over the neck and trunk region for 1 day. The lesions appeared early in the morning and were initially pin head size which later disseminated to the chest, limbs and almost 80-90% of her body area symmetrically with fluid-filled lesions (blisters) which then coalesced to form bullae and started to peel off slowly within 2 days of onset. She also had a gritty sensation and redness of her eyes before the appearance of the rash. Later that day she also had painful oral lesions causing difficulty in swallowing. There was a history of pain in over left foot 1 month back for which she was treated with etoricoxib. She had a similar illness in the past and also gave a history of Raynaud's phenomenon of exposure to cold. She had no history of fever, dyspnea, cough, abdominal pain, burning micturition, hematuria, previous drug reactions and allergies, no associated malignancies, or weight loss. There was no significant family history. The patient denied any use of alcohol, smoking and illicit drugs.

**Figure 1:**
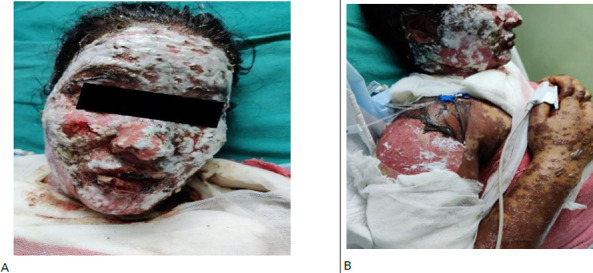
A) Hemorrhagic crusts seen over lips and topical steroid cream applied to denuded areas of the face, B) Fluid-filled lesions with peeling off of the skin of upper limbs.

On examination, her vitals were normal and bilateral pedal oedema was present. Multiple purpuric, erythematous lesions were present over her face, scalp, chest, trunk, abdomen, genitals and legs. Multiple erosions and hemorrhagic crusts were also present over her lips and oral mucosa (Figure 1).

**Figure 2:**
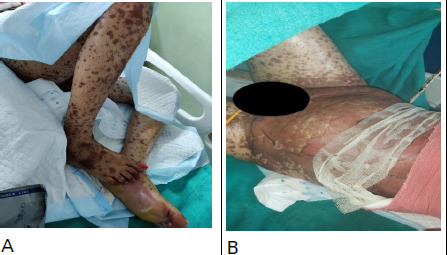
A) Hyperpigmented macular skin lesions spreading to lower limbs, B) Skin lesions spreading to abdomen and genitalia involving >90% of BSA.

Congestion and symblepharon of both eyes were noted. Eroded skin with an erythematous base was present on the genital area. Nikolsky sign was positive, tenderness was present and >90% body surface area was involved (Figure 2).

On systemic examination, no significant findings were noted. Laboratory investigations showed lymphopenia (2700/cubic mm), haemoglobin (11.5 gm%), hypokalemia (3.2 meq/l), hypoalbuminemia (2.5 gm/ dl), increased C-reactive protein (286 mg/l), elevated Anti-nuclear antibody level (8.46), Anti-SSA (anti-Sjogren's-syndrome-related antigen A autoantibodies) antibody (+++), Anti- Ro antibody (+++), random blood sugar (130 mg/dl), liver function test was normal, serology for Human immunodeficiency virus (HIV) was negative. Chest X-Ray Posterior-Anterior view showed no significant findings. She was immediately shifted to the intensive care unit and treated symptomatically.

There was a multidirectional treatment approach including replacement of fluid loss and maintaining electrolyte balance. Further, treatment was done with intravenous (IV) dexamethasone 8 mg daily for 10 days. Besides, an antibiotic treatment with ceftriaxone 1 gm IV was also given twice a day for 10 days along with 100 ml 20% human albumin supplementation over 8-10 hours for 3 days. The wet dressing was done every alternate day. Pain management was done with IV tramadol. Symblepharon from both eyes was removed and ciprofloxacin and refresh tears eye drops were given. Treatment for wound care was done with topical steroid therapy (fusid-b cream), paraffin and cloderm cream. Re-epithelization started at about 2 weeks. The wound swab culture was negative and the patient started recovering gradually. So, the dose of dexamethasone and antibiotic was tapered appropriately. The patient was discharged after 20 days on oral prednisolone, an oral antibiotic, candid mouth paint, topical steroid cream (fusid-b) and placentrix cream.

## DISCUSSION

TEN is one of the severest immunologically-mediated adverse drug reactions which is considered to be a type IV hypersensitivity reaction, primarily T-cell mediated. The incidence of Systemic lupus erythematosus (SLE) in the Asia Pacific region is 0.9-3.1 per 100000 population.^4^ Although the incidence of TEN in SLE has been reported, a study found that individuals with SLE had an odds ratio close to one for TEN.^5^

Although the exact aetiology of TEN is not known, it is usually due to drug reaction with or without associated infection and malignancy.^6^ Many recent studies have also shown an association between Human Leukocyte Antigen alleles and the development of TEN, mostly in the Southeast Asian population.^7-9^ Although TEN is a rare disease, an association of TEN with SLE is even rarer.^10^ There is a female predilection with a female to male ratio of 1.5 to 1.11 The major causative drugs for TEN were antimicrobials (37.27%), fluoroquinolones (8.48%), anti-tubercular (5.65%), penicillins (5.39%), cephalosporins (3.08%), anti-epileptics (35.73%) and NSAIDs (15.93%), carbamazepine (18.25%), phenytoin (13.37%) and paracetamol (6.17%).^12,13^ Duration between exposure to drug and exacerbation of reaction ranges from few hours to days.^14^

In this case, our patient has developed gritty and burning sensations in the eyes, oral mucosal lesions, and maculopapular erythematous itchy rash within 10 days of drug intake which was a prodromal phase. Within the next 2 days, rashes turned into blisters then bullae peeling off of the necrotic skin extending to the scalp, face, trunk, extremities and also genito-urinary area. This patient was under Etoricoxib for 7 days, so the probable cause for the development of TEN could be Etoricoxib and SLE was a cofactor. Based on the laboratory findings and Systemic Lupus International Collaborating Clinics (SLICC) criteria done in our setting, she was diagnosed with SLE fulfilling four criteria with three clinical and one immunologic criterion.^15^ A disease severity score was used to predict the outcome of the TEN. In this case, the score obtained disease severity score for Toxic Epidermal Necrolysis (SCORTEN) was two with a predicted mortality rate of 12%.^16^,^17^

The treatment with IV fluid, IV Dexamethasone, a topical steroid and regular wet dressing along with IV ceftriaxone, a broad spectrum antibiotic was found to be effective in this case. No exact treatment except symptomatic management is yet known to benefit the patients of TEN.^18^ Even though the incidence of TEN in the case of SLE is very rare, it was a case of Etoricoxib-induced Toxic Epidermal Necrolysis in a patient with SLE.

## References

[1] Roujeau JC, Stern RS (1994). Severe adverse cutaneous reactions to drugs.. N Engl J Med..

[2] Sehgal VN, Srivastava G (2005). Toxic epidermal necrolysis (TEN) Lyell's syndrome.. J Dermatolog Treat..

[3] Li LF, Ma C (2006). Epidemiological study of severe cutaneous adverse drug reactions in a city district of China.. Clin Exp Dermatol..

[4] Kafle MP, Lee V (2016). Systemic lupus erythematosus in Nepal: A review.. Lupus..

[5] Hsu DY, Brieva J, Silverberg NB, Silverberg JI (2016). Morbidity and Mortality of Stevens-Johnson Syndrome and Toxic Epidermal Necrolysis in United States Adults.. J Invest Dermatol..

[6] Yetiv JZ, Bianchine JR, Owen JA (1980). Etiologic factors of the Stevens-Johnson syndrome.. South Med J..

[7] Locharernkul C, Loplumlert J, Limotai C, Korkij W, Desudchit T, Tongkobpetch S (2008). Carbamazepine and phenytoin induced Stevens-Johnson syndrome is associated with HLA-B*1502 allele in Thai population.. Epilepsia..

[8] Cheung YK, Cheng SH, Chan EJ, Lo SV, Ng MH, Kwan P (2013). HLA-B alleles associated with severe cutaneous reactions to antiepileptic drugs in Han Chinese.. Epilepsia..

[9] Hung SI, Chung WH, Liou LB, Chu CC, Lin M, Huang HP (2005). HLA-B*5801 allele as a genetic marker for severe cutaneous adverse reactions caused by allopurinol.. Proc Natl Acad Sci U S A..

[10] Fan WY, Zhai QR, Ma QB, Ge HX (2022). Toxic epidermal necrolysis with systemic lupus erythematosus: case report and review of the literature.. Ann Palliat Med..

[11] Mockenhaupt M (2017). Epidemiology of cutaneous adverse drug reactions.. Allergol Select..

[12] Patel TK, Barvaliya MJ, Sharma D, Tripathi C (2013). A systematic review of the drug-induced Stevens-Johnson syndrome and toxic epidermal necrolysis in Indian population.. Indian J Dermatol Venereol Leprol..

[13] Acharya A, Acharya SP, Bhattarai TR (2020). Cotrimoxazole Induced Steven Johnson Syndrome: A Case Report.. JNMA J Nepal Med Assoc..

[14] Ellender RP, Peters CW, Albritton HL, Garcia AJ, Kaye AD (2014). Clinical considerations for epidermal necrolysis.. Ochsner J..

[15] Oku K, Atsumi T, Akiyama Y, Amano H, Azuma N, Bohgaki T (2018). Evaluation of the alternative classification criteria of systemic lupus erythematosus established by Systemic Lupus International Collaborating Clinics (SLICC).. Mod Rheumatol..

[16] Bastuji-Garin S, Fouchard N, Bertocchi M, Roujeau JC, Revuz J, Wolkenstein P (2000). SCORTEN: a severity-of-illness score for toxic epidermal necrolysis.. J Invest Dermatol..

[17] Kameshwari JS, Devde R (2015). A case report on toxic epidermal necrolysis with etoricoxib.. Indian J Pharmacol..

[18] Micheletti RG, Chiesa-Fuxench Z, Noe MH, Stephen S, Aleshin M, Agarwal A (2018). Stevens-Johnson Syndrome/Toxic Epidermal Necrolysis: A Multicenter Retrospective Study of 377 Adult Patients from the United States.. J Invest Dermatol..

